# Does Physical Activity Increase Life Expectancy? A Review of the Literature

**DOI:** 10.1155/2012/243958

**Published:** 2012-07-01

**Authors:** C. D. Reimers, G. Knapp, A. K. Reimers

**Affiliations:** ^1^Klinik für Neurologie, Zentralklinik Bad Berka, Robert-Koch-Allee 9, 99438 Bad Berka, Germany; ^2^Fakultät Statistik, Technische Universität Dortmund, 44221 Dortmund, Germany; ^3^Sportwissenschaft, Universität Konstanz, Universitätsstraße 10, 78457 Konstanz, Germany

## Abstract

Physical activity reduces many major mortality risk factors including arterial hypertension, diabetes mellitus type 2, dyslipidemia, coronary heart disease, stroke, and cancer. All-cause mortality is decreased by about 30% to 35% in physically active as compared to inactive subjects. The purpose of this paper was to synthesize the literature on life expectancy in relation to physical activity. A systematic PubMed search on life expectancy in physically active and inactive individuals was performed. In addition, articles comparing life expectancy of athletes compared to that of nonathletes were reviewed. Results of 13 studies describing eight different cohorts suggest that regular physical activity is associated with an increase of life expectancy by 0.4 to 6.9 years. Eleven studies included confounding risk factors for mortality and revealed an increase in life expectancy by 0.4 to 4.2 years with regular physical activity. Eleven case control studies on life expectancy in former athletes revealed consistently greater life expectancy in aerobic endurance athletes but inconsistent results for other athletes. None of these studies considered confounding risk factors for mortality. In conclusion, while regular physical activity increases life expectancy, it remains unclear if high-intensity sports activities further increase life expectancy.

## 1. Introduction

The most important causes of death in Western industrialized countries are cardio- and cerebrovascular diseases and malignancies. For instance, in Germany in 2008, 68.6% of all women and 65.9% of all men died from these diseases. In contrast, the third most frequent cause of death are respiratory diseases which cause less than 10% of deaths each year (Table 1). Important risk factors for cardio- and cerebrovascular diseases include smoking, arterial hypertension, obesity, diabetes mellitus, and dyslipidemia along with atrial fibrillation for ischemic strokes [1, 2].

Regular physical activity reduces the risk of and/or improves many diseases and conditions including arterial hypertension, diabetes mellitus type 2, dyslipidemia, obesity, coronary heart disease, chronic heart failure [3, 4], and chronic obstructive pulmonary disease [4]. In addition, the risk of colon [3, 5], breast [4, 5], and possibly endometrial, lung, and pancreatic cancer is reduced [5] (Table 2).

The relative risk of death is approximately 20% to 35% lower in physically active and fit persons compared to that in inactive and unfit persons [6, 7]. Physical inactivity represents a major independent risk factor for mortality accounting for up to 10% of all deaths in the European region [3]. Hence, because a 40% lower mortality rate corresponds to an approximately 5-year higher life expectancy [8], one would expect an approximately 3.5- to 4.0-year higher life expectancy in physically active persons compared to that in inactive persons.

The purpose of this review was to synthesize the literature on life expectancy in relation to physical activity. Specifically, cohort studies on physically active and inactive subjects were reviewed to detect a possible difference in life expectancy between these subject groups. In addition, cohort studies on athletes and non-athletes were reviewed to detect a possible difference in life expectancy between these subject groups.

## 2. Method

To identify all relevant articles about cohort studies investigating the life expectancy of physically active versus inactive persons, a systematic literature search was conducted in the electronic bibliographical database PubMed (http://www.ncbi.nlm.nih.gov/pubmed/). We only searched for English-language peer-reviewed journal articles using the search terms “(life expectancy OR longevity) AND (physical activity OR exercise OR sport)” (last update January 3, 2012). A total of 1,932 articles were found. Using the abstracts and/or titles, forty-one of these articles were identified as cohort studies comparing mortality and/or life expectancy of physically active and inactive persons. However, of these, only 13 articles presented detailed data on life expectancy for both groups.

Subsequently, a search was performed using the terms “(life expectancy OR longevity) AND athlete” to find articles on life expectancy in (former) athletes. Sixty-six articles were found. Additional publications were identified using the search term “(life expectancy OR longevity) AND (physical activity OR exercise OR sport).” Of all these articles, 21 articles investigated mortality and/or life expectancy of (former) athletes. Eleven of these articles presented detailed data on life expectancy of athletes compared to that of a control group.

The remaining life expectancy for physically active and inactive individuals or the difference in remaining life expectancy between the two groups, respectively, were reported in the articles. However, the attained ages of subject groups differed between studies. Because the differences of remaining life expectancies cannot be assumed to be independent from the attained age, we report the results stratified for attained age. Within each stratum of attained age, the results were presented for both sexes.

Only five articles [16–20] reported confidence intervals for the difference in remaining life expectancies between groups. In four articles [16, 18–20], a parametric bootstrap procedure was used, and in one publication [17] a nonparametric maximum likelihood approach was used. Consequently, we did not combine the results in a meta-analysis model.

Because a meta-analysis model was not appropriate, all data found in the literature search were reported despite some overlaps between the cohort studies. For instance, Jonker et al. [18] and Nusselder et al. [19] used data from the Framingham Heart Study and Paffenbarger et al. [21–24] used data from the Harvard Alumni cohort. While most studies reported results based on multivariate life-table analysis, the studies by Menotti et al. [25] and Pekkanen et al. [26] only reported the results of gained life expectancies for the cohorts based on classical survival analysis. Additionally, the study of Pekkanen et al. [26] reported the survival rate of men aged 40 to 65 years in the following 20 years and not the total life expectancy.

## 3. Results

Thirteen cohort studies presented data on life expectancy in physically active individuals compared to that in physically inactive control subjects (Table 3). All studies reported a higher life expectancy in physically active subjects, ranging from 0.43 to 6.9 additional years (mean ± one standard deviation, men: 2.9 ± 1.3 years, women: 3.9 ± 1.8 years). Eleven studies considered confounding factors that could affect life expectancy, such as body mass index, blood pressure, diabetes mellitus, dyslipidemia, cardiovascular and lung diseases, cancer, smoking, or alcohol consumption [16–25, 27]. The additional life expectancy in physically active compared to inactive persons in these studies ranged between 0.43 and 4.21 years (2.7 ± 1.1 years). The physically most active groups, included in the estimations of life expectancy, participated in moderate to high leisure time [16, 27–29] or leisure time and all-day activities [17–25].

The median increase of life expectancy of men and women in the eight studies presenting data on both sexes amounted to 3.7 years each. Physical activity during leisure time seems to increase life expectancy more effectively than total physical activity (all-day, professional, or leisure time activity altogether; professional physical activity alone has not been studied): 3.4 added years due to total activities and 4.7 added years (median values) due to leisure time activities in women, 1.9 and 3.9 added years, respectively, in men. The number of studies, however, is too low for a statistical analysis. Furthermore, the description of the amount of physical activity in the active and inactive groups are too heterogeneous for any statistical correlation between the amount of activity and the added years of life.

The eleven case control studies on life expectancy of athletes, mostly elite athletes (Table 4), reported a mean life expectancy that was between 5.0 years lower and 8.0 years higher than that of the nonathlete control groups. Aerobic endurance sports resulted on average in a 4.3 to 8.0 years higher life expectancy and team sports activities on average in a 5.0 years lower to about 5 years higher life expectancy compared to that for normal physical activity. Only one study presented data on strength sports and reported a slightly higher life expectancy compared to that for normal physical activity. None of these studies considered any confounding factor that could affect life expectancy.

## 4. Discussion

The purpose of this review was to synthesize the literature on life expectancy in relation to physical activity. Being physically active indeed appears to be associated with a higher life expectancy. Samitz et al. [7] as well as Warburton et al. [11] reported a mean reduction of mortality of 31% to 35% in persons who participate in regular leisure-time or daily life physical activity compared to that in inactive persons. Assuming a 40% lower mortality corresponding to a 5-year higher life expectancy [8], regular physical activity should increase mean life expectancy by approximately (31% to 35%)/40% × 5 years or by 3.9 to 4.4 years. Indeed, the few articles that presented data on life expectancy in physically active individuals reported a 0.43- to 6.9-year higher life expectancy. High-quality studies considering confounding factors that could affect mortality reported a 0.43- to 4.21-years higher life expectancy in physically active compared to inactive persons. The wide range of years added cannot be explained based on the published data.

The studies that standardized extended life estimates for confounding factors [16, 18–25, 27] virtually calculated a net gain in life expectancy by being physically active. However, the actual increase in life expectancy should be much higher because of favorable effects of physical activity on other risk factors for mortality such as arterial hypertension, glucose and lipid metabolism, coronary heart disease, stroke, or malignancies (Table 1). In fact, nonsmoking, normal weight, and physically fit men live on average 12 years (95% confidence interval, 8.6 to 14.6 years) longer than smoking, overweight, and physically unfit control subjects [41]. Subjects who never smoked, follow a healthy diet, are adequately physically active, and consume only moderate alcohol have a mean life expectancy that is 11.1 years longer than those who practice none of these healthy life behaviors [42].

The mechanisms underlying the net effect of physical activity are speculative and include reduction of triglyceride and apolipoprotein B concentrations, increase of high-density lipoproteins and tissue plasminogen activator activity, and reduction of coronary artery calcium resulting in reduced risks of vascular diseases, which carry the strongest mortality risk [43]. In addition, regular physical activity increases the endurance of cells and tissues to oxidative stress, vascularization, and energy metabolism [44].

According to the results of the meta-analysis on all-cause mortality in relation to physical activity performed by Samitz et al. [7], vigorous physical activity (>6 metabolic equivalents (MET)) reduces mortality slightly, but this reduction is significantly more pronounced than that for moderate activity (3–6 MET).

A greater life expectancy is not associated with more years of being frail and depending on assistance. In contrary, Nusselder et al. [20] reported a gain of disability-free years of life with a higher life expectancy.

The few data available on life expectancy in athletes who were much more physically active than the average individual are inconclusive. All studies proved an increased life expectancy in endurance athletes ranging between 2.8 to 8.0 added years. This gain is probably higher than that found for persons performing vigorous physical activity in the cohort studies. In team sports and other sports disciplines, life expectancy may fall below or be above that of the control groups. However, data on health behaviors of these athletes other than their physical activity during their active sports career such as smoking, food, and alcohol consumption are not available. Thus, the effect of elite sports activities on life expectancy warrants further investigation.

In summary, as expected from numerous prospective cohort studies on all-cause mortality in physically active and physically inactive persons, estimates on life expectancy in relation to physical activity indicate additional years of life in active subjects: the conservative estimate of the net increase in life expectancy with physical activity is about 2–4 years but presumably even greater because of the positive influence of physical activity on major risk factors for mortality.

## Figures and Tables

**Table 1:** Number of deaths (percentage of total number of deaths) for the three most frequent causes of death for women and men in Germany in 2008 [9].

	Women (per 100,000 persons/y)	Men (per 100,000 persons/y)
Total	467.3 (100.0%)	720.5 (100.0%)

Vascular diseases	186.4 (39.9%)	263.6 (36.6%)
Coronary heart disease	61.8 (13.2%)	117.4 (16.3%)
Strokes	35.8 (7.7%)	41.6 (5.8%)
Malignant tumors	134.0 (28.7%)	210.9 (29.3%)
Respiratory diseases	27.7 (5.9%)	53.3 (7.4%)

**Table 2:** Preventive effects of regular physical activity on major risk factors for cardio- and cerebrovascular diseases and cancer.

Author(s)	Risk factor	Effect of regular physical activity on the risk factor in healthy subjects
Adami et al. [3], Halle and Schoenberg [10], Warburton et al. [6], Warburton et al. [11]	Colon cancer	Incidence −30% to −40%
Adami et al. [3], Monninkhof et al. [12], Warburton et al. [6]	Breast cancer	Incidence −20% to −50%
Walker et al. [13], Warburton et al. [11]	Type 2 diabetes mellitus	Incidence −28% to −59%
G. A. Kelley and K. S. Kelley [14]	Dyslipidemia	HDL cholesterol +11%
Pedersen and Saltin [4]	Arterial hypertension	Systolic and diastolic blood pressure −3.84/−2.58 mmHg
Pedersen and Saltin [4]	Obesity	Increased chance to maintain body weight
Warburton et al. [11], Reimers et al. [15]	Stroke	Incidence −27% to −40%

**Table 3:** Cohort studies comparing the life expectancy of physically active and inactive persons.

Sex	Age (class) at start of followup	Authors	Country	Number of individuals, duration of followup	Estimate additional years of life (95% CI) (years)	Activity of the “active” group	Activity of the “inactive” group	Confounding factors
Women	30	Fraser and Shavlik [17]	USA	^ #^12 y	2.19 (0.92–3.45)^∗^	At least 3 times per week vigorous all-day or sports activities ≥15 min.	Less than 3 times per week intensive all-day and sportive activities ≥15 min.	Vegetarian/nonvegetarian, high/low nut consumption, body mass index, never/past smoker, hormone replacement therapy
	30	Wen et al. [27]	Taiwan	216.910 8.05 ± 4.02 y	3.67 84.08 versus 87.75	Very vigorous physical activity (≥25.5 MET·h/week) during leisure time	Inactivity (<3.75 MET·h/week) during leisure time	Age, sex, education, physical work, smoking, alcohol consumption, diabetes mellitus, arterial hypertension, cancer, fasting glucose, systolic blood pressure, total cholesterol, body mass index
	45	Bélanger et al. [28]	Canada	^ #^2 y	6.9 81.7 versus 88.6	≥1.5 kcal/kg/d energy expenditure during leisure time	<1.5 kcal/kg/d energy expenditure during leisure time	
	50	Jonker et al. [18]	USA (Framingham Heart Study)	2.813 12 y	3.7 (2.6–4.9) 82.3 versus 86.0	High physical activity level (>33 METs/d)	Low physical activity level (<30 METs/d)	Age, education, smoking, marital status, cardiovascular and lung diseases, cancer, left ventricular hypertrophy, arthritis, ankle edema, total cholesterol, familial history of diabetes mellitus
	50	Nusselder et al. [20]	The Netherlands	1.447 2 y	1.8 (0.5–2.7) 76.5 versus 78.3	Walking, biking, gardening, sports >17.33 METs/week	Walking, biking, gardening, sports <12 METs/week	Age, sex, education, cardiovascular disease, cancer, COPD, arthritis, back complaints, neurological diseases
	50	Nusselder et al. [19]	USA (Framingham Heart Study)	2.873 12 y	3.4 (2.3–4.5) 82.7 versus 86.1	High physical activity level (>33 METs/d)	Low physical activity level (<30 METs/d)	Age, sex, education, marital status, smoking, body mass index, blood pressure, cancer, diabetes mellitus, left ventricular hypertrophy, ankle edema, any pulmonary disease, smoking
	65	Ferrucci et al. [29]	USA	5.215 6 y	Nonsmoker: 5.7 77.7 versus 83.4 Smoker: 4.2 76.1 versus 80.3	High physical activity (gardening, walking, vigorous exercise) each once per week or several times per month *or* one of these activities several times per week and another activity once per week or several times per month	Activities as in the active group at most once per month	

Men	30	Fraser and Shavlik [17]	USA	^ #^12 y	2.1 (0.4–3.9)^∗^	At least 3 times per week vigorous all-day or sports activities ≥15 min.	Less than 3 times per week intense all-day and sportive activities ≥15 min.	Vegetarian/nonvegetarian, high/low nut consumption, body mass index, never/past smoker, hormone replacement therapy
	30	Wen et al. [27]	Taiwan	199.265 8.05 ± 4.02 y	4.21 80.37 versus 84.58	Very vigorous physical activities (≥25.5 MET·h/week) during leisure time	Inactivity (<3.75 MET·h/week) during leisure time	Age, sex, education, physical work, smoking, drinking, diabetes mellitus, arterial hypertension, history of cancer, fasting blood glucose, systolic blood pressure, total cholesterol, body mass index
	35–39	Paffenbarger et al. [21]	USA (Harvard Alumni)	16.936 16 y	1.5^∗^	Physical activities (walking, climbing stairs, sports) ≥2.000 kcal/week	Physical activities (walking, climbing stairs, sports) <2.000 kcal/week	Age, cigarette smoking, arterial hypertension, body mass index, age of parental death
	35–39	Paffenbarger et al. [22]	USA (Harvard Alumni)	16.936 12–16 y	2.51^∗^	Physical activities (walking, climbing stairs, sports) ≥2.000 kcal/week	Physical activities (walking, climbing stairs, sports) <500 kcal/week	Age, cigarette smoking, arterial hypertension, body mass index, age of parental death
	40–59	Menotti et al. [25]	Italy	1.712 40 y	1.6^∗^	Physically active	Sedentary	Age, family history of both parents, mean blood pressure, serum cholesterol, mid-arm circumference, forced exspiratory volume, chronic diseases (cardiovascular, diabetes mellitus, cancer), corneal arcus, xanthelasma, body mass index
	45	Bélanger et al. [28]	Canada	^ #^2 y	3.9 76.9 versus 80.8	≥1.5 kcal/kg/d energy expenditure during leisure time	<1.5 kcal/kg/d energy expenditure during leisure time	
	45–54	Paffenbarger et al. [23]	USA (Harvard Alumni)	10.269 8 y	0.43^∗^	Physical activities ≥2.000 kcal/week	Physical activities <2.000 kcal/week	Age, cigarette smoking, arterial hypertension, overweight, early parental death
	45–54	Paffenbarger et al. [24]	USA (Harvard Alumni)	14.785 11 y	1.78^∗^	Physical activity (walking, stair climbing, sports, or recreational activities) increased from <1.500 to ≥1.500 kcal/week	Physical activity (walking, stair climbing, sports, or recreational activities) continuing <1.500 kcal/week	Age, cigarette smoking, arterial hypertension, overweight, alcohol consumption, early parental death, chronic diseases
	50	Jonker et al. [18]	USA (Framingham Heart Study)	2.396 12 y	4.1 (2.8–5.4) 75.3 versus 79.4	High physical activity level (>30 METs/d)	Low physical activity level (<30 METs/d)	Age, education, smoking, marital status, cardiovascular and lung diseases, cancer, left ventricular hypertrophy, arthritis, ankle edema, total cholesterol, familial history of diabetes mellitus
	50	Nusselder et al. [20]	The Netherlands	1.519 2 y	2.9 (0.9–4.3) 74.8 versus 76.7	Walking, biking, gardening, sports >17.33 METs/week	Walking, biking, gardening, sports <12 METs/week	Age, sex, education, cardiovascular disease, cancer, chronic obstructive pulmonary disease, arthritis, back complaints, neurological diseases
	50	Nusselder et al. [19]	USA (Framingham Heart Study)	2.336 12 y	3.5 (2.5–4.6) 76.4 versus 80.0	High physical activity level (>33 METs/d)	Low physical activity level (<30 METs/d)	Age, sex, education, marital status, body mass index, blood pressure, cancer, diabetes mellitus, left ventricular hypertrophy, ankle edema, any pulmonary disease, smoking
	50	Byberg et al. [16]	Sweden	2.205 35 y	2.3 (1.3–3.3)^∗^	Regularly hard physical training or competitive sport or any active recreational sports or heavy gardening at least 3 hours every week	Spending most of the time reading, watching TV, going to the cinema, or engaging in other, mostly sedentary activities	Smoking, weight and height, alcohol use, obesity, diabetes mellitus, musculoskeletal, neurological, or psychiatric disorders, blood pressure, antihypertensive drugs, total serum cholesterol, educational level, socioeconomic group
	65	Ferrucci et al. [29]	USA	3.389 6 y	Nonsmoker: 5.2 76.0 versus 81.2 Smoker: 3.4 74.5 versus 77.9	High physical activity (gardening, walking, vigorous exercise) each once per week or several times per month *or* one of these activities several times per week and another activity once per week or several times per month	Activities as in the active group maximally once per month	

^∗^Total life expectancy not presented, ^#^number of individuals not differentiated between men and women.

**Table 4:** Case control studies presenting life expectancy of (former) athletes compared to that of control subjects.

Author(s)	Type of sports	Reduction/increase in life expectancy (y)
Prout [30]	Endurance sports (college rowers from Harvard and Yale)	+6.3

Sarna et al. [31]	Endurance sports (long distance running, cross-country skiing)	+5.7

Karvonen [32], Karvonen et al. [33]	Endurance sports (cross-country skiing)	+2.8 to +4.3

Sanchis-Gomar et al. [34]	Endurance sports (Tour de France cyclists)	+8.0

Sarna et al. [31], Sarna and Kaprio [35]	Power sports (throwing sports, wrestling, weight lifting, boxing)	+1.6

Sarna et al. [31], Sarna and Kaprio [35]	Team sports (ice hockey, soccer, basketball, other outdoor sports)	+4.0

Abel and Kruger [36]	Team sports (baseball)	−5.0

Abel and Kruger [37]	Team sports (baseball)	+4 to 5

Kuss et al. [38]	Team sports (German international soccer players)	−1.9 J. (+0.6 to −3.2)

Hudec et al. [39]	Various sports disciplines	−0.38

Rook [40]	Various sports disciplines	+1.03

## Abbreviations

CI: Confidence interval
COPD: Chronic obstructive pulmanry disease
d: Day
h: Hour(s)
MET: Metabolic equivalent value (1 MET = 1 kcal per h per kg of bodyweight)
Min.: Minute(s)
TV: Television
vs: Versus
y: Year(s).
